# The Hyperarid Core of the Atacama Desert, an Extremely Dry and Carbon Deprived Habitat of Potential Interest for the Field of Carbon Science

**DOI:** 10.3389/fmicb.2017.00993

**Published:** 2017-06-08

**Authors:** Armando Azua-Bustos, Carlos González-Silva, Gino Corsini

**Affiliations:** ^1^Centro de Astrobiología (Consejo Superior de Investigaciones Científicas-Instituto Nacional de Técnica Aeroespacial)Madrid, Spain; ^2^Instituto de Ciencias Biomédicas, Facultad de Ciencias de la Salud, Universidad Autónoma de ChileSantiago, Chile; ^3^Centro de Investigación del Medio Ambiente, Universidad Arturo PratIquique, Chile

**Keywords:** Atacama Desert, organic carbon, desert ecosystems, Mars, carbon science

## Abstract

The Atacama Desert in Chile is the driest and oldest desert on Earth, also considered one of the best Mars analog models. Here, several heterotrophic microbial communities have been discovered in its driest regions, with the ones present in the soil subsurface being one of the most interesting due to its existence in a habitat with almost no water available and almost undetectable organic carbon sources. Our recent discovery of the driest site of the Atacama known to date (and the heterotrophic microbial species that are able to survive in this site) reaffirms the opportunity to better characterize the physiological and molecular mechanisms that these species use to detect, mobilize, incorporate and use carbon under these extremely harsh conditions. Here we summarize what has been reported up to date on the organic carbon concentrations in different sites of the hyperarid core of the Atacama Desert, proposing that due to the meager amounts of carbon and extremely dry conditions, the microbial communities of the hyperarid core of the Atacama Desert may be of interest for the field of carbon science.

## Introduction

**FIGURE 1:**
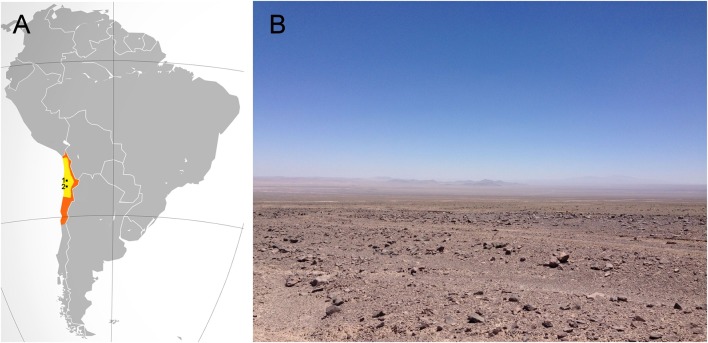
The hyperarid core of the Atacama Desert. **(A)** In orange, the location of the Atacama Desert. In yellow, the hyperarid core of the Atacama. The small black dots show the relative locations of Maria Elena South (1) and Yungay (2). **(B)** A typical view of the hyperarid core of the Atacama near the María Elena site.

The Atacama Desert, located in northern Chile (**Figure 1**), encompasses about 105.000 square kilometers. It is bordered on the east by the Andes Mountains and on the west by the Coastal Range. The Atacama is well known for being the driest (McKay et al., 2003; Azua-Bustos et al., 2015) and oldest desert on Earth, estimated to be arid of the past 150 million years and hyperarid for the past 15 million years (Pueyo et al., 2001; Houston and Hartley, 2003; Hartley et al., 2005; Rech et al., 2006).

It was thought that no lifeforms could survive the extremely dry a high UV radiation levels of the hyperarid core of the Atacama (Navarro-González et al., 2003), however, several heterotrophic microbial species were later reported living in this extremely harsh environment (reviewed in Azua-Bustos et al., 2012a). Although the current interest of the scientific community is to understand how these microorganisms are able to tolerant the extreme desiccation and high UV radiation conditions of the Atacama (Wierzchos et al., 2006; Azua-Bustos et al., 2009, 2010, 2011; Paulino-Lima et al., 2016), nothing has yet been reported on how heterotrophic species are able to obtain, mobilize and use organic carbon in a place where carbon and water are virtually absent. In this short hypothesis paper, we review the reports up to date on organic carbon concentrations in soils of the Atacama, proposing that due to the meager amounts of organic carbon and extremely dry conditions, the microbial communities of the hyperarid core of the Atacama Desert may be of interest for the field of carbon science (for a review on the biodiversity of heterotrophic microbial species reported in the hyperarid core of the Atacama see Azua-Bustos et al., 2012a,b).

## What has been Reported about Organic Carbon and Life in the Atacama Desert

Navarro-González et al. (2003) were the first to report organic carbon concentrations of soils of the hyperarid core of the Atacama. Using samples from the Yungay region, these authors used Pyrolysis-Gas Chromatography-Mass Spectrometry to report that soil samples from the Yungay region were almost depleted of organic molecules, with only minor amounts of formic acid (1 mmol g^-1^) and benzene (1 mmol g^-1^), compounds typically released by the thermal treatment of various types of organic molecules. The formic acid/benzene ratio suggested that the organic matter present in this region was highly oxidized and probably composed of refractory organics such as aliphatic and aromatic mono/polycarboxylic acids. Interestingly, these authors reported that samples from the Yungay region contained extremely low levels of heterotrophic bacteria detected by dilution plating (only one colony per every 10 plates), with no DNA recovered from these soils.

Later, Glavin et al. (2004) using a new method to estimate bacterial cell counts analyzed surface and subsurface samples from the Flat Top Hill site, located approximately 12 km south east of Yungay. These authors found only trace levels of nucleobases (0.04–0.6 nmol/g) in subsurface soil samples, corresponding to a total bacterial concentration of 4.4 × 10^6^ of *Escherichia coli* cell equivalents (ECE)/g of soil. In the case of the superficial samples, no adenine was detected, suggesting levels below 5 × 10^6^ ECE/g.

In a later report, Glavin et al. (2006) analyzed surface soil samples from Yungay, finding adenine concentrations of 0.04 nmol/g corresponding to about 4 × 10^6^ *E. Coli* cell equivalents/g. This value was two orders of magnitude higher than total viable counts of culturable bacteria previously measured by serial dilution plating in Yungay samples by Navarro-González et al. (2003) suggesting that these soil samples contained mostly non-culturable bacteria.

Buch et al. (2006) using a novel extraction procedure coupled with chemical derivatization to target organic compounds by gas chromatography mass spectrometry (GC-MS) also analyzed soil samples from the hyperarid core. GC-MS analysis of water and isopropanol (1:1 mixture) extracts showed that both amino (alanine; 1.1 × 10^-9^) and carboxylic acids (benzoic acid; 2.7 × 10^-9^) were readily extracted, supporting the hypothesis of Benner et al. (2000) in that carboxylic acids such as benzenecarboxylic acid are abundant in oxidizing soils like the ones found in the Atacama, as well as other organic compounds of biological importance like urea and amino acids.

Amashukeli et al. (2007) also addressed the organic composition and oxidation chemistry of soils of the Yungay region. Using soil surface samples, amino acids like glycine, alanine, valine, aspartic acid, serine and glutamic acid were readily extracted in the range of 1–70 ppb. The number of cell equivalents in one gram of soil calculated from this data was estimated to be slightly higher (10^5^) than the ones previously reported for surface samples, owing to the alleged ability of their method to extract amino acids from both viable and non-viable cells.

Lester et al. (2007) also analyzed surface and subsurface soil samples of the Yungay region, finding extremely low total organic carbon contents, ranging between 560 and 765 mg/g. PLFA analysis unveiled surface concentrations of about 1.0 × 10^7^ cells/g, five times higher than that of subsurface samples, which ranged from 2.0 × 10_6_ to 2.4 × 10_6_ cells/g.

Connon et al. (2007) similarly analyzed surface and subsurface soil samples in the Yungay area, focusing in two sites that were previously analyzed by Navarro-González et al. (2003) and Lester et al. (2007). These authors found that the total organic carbon content of these soils was extremely low, ranging between 200 and 700 mg per g of soil. PLFA analysis showed values ranging from 2 × 10^5^ to 7 × 10^6^ cell equivalents per gram of soil.

Barros et al. (2008) also examined soils samples from the hyperarid core of the Atacama by using Calorimetry, reporting that total carbon in these soils ranged from 0.17 to 2.66 g per 100 g of soil, with the lowest values measured at the driest sites.

In one of the most focused studies on this topic, Fletcher et al. (2012) investigated the variability of surface soil organic carbon within the Yungay region. They reported labile organic carbon values that ranged from 2 to 73 mg/g of soil, consistent with the variability of other studies in the Yungay area. Crits-Christoph et al. (2013) also analyzed samples from six locations in the hyperarid core, reporting TOC values of less than 0.01%.

In 2015 we reported the discovery of Maria Elena South (22°15′39′′S, 69°43′29′′W) the driest site of the already hyperarid Atacama (Azua-Bustos et al., 2015). We found that soil samples from Maria Elena South showed extremely low, albeit variable levels of organics (0.1–1.1%. Depending on the depth from where samples were taken, we proposed that this variability may represent the number of microbial species (*Streptomyces, Bacillus, Geodermatophilus*) found along the soil profile.

In an interesting approach to analyze microhabitats in the hyperarid core of the Atacama, Warren-Rhodes et al. (2006) examined the occurrence of heterotrophic and autotrophic hypolithic microorganisms colonizing translucent stones along an aridity gradient. These authors found that total organic carbon was five times more in the soil under the hypolithic community (46 ± 15 mmol g^-1^) than in the surface soil (9 ± 3 mmol g^-1^), suggesting that these colonized stones are extreme types of “islands of fertility” in the Atacama.

In a similar approach toward microhabitats colonized by heterotrophic and autotrophic microorganisms, Ziolkowski et al. (2013) reported total organic carbon concentrations in samples from gypsum, Ignimbrite, and halites from a salt pan close to the Coastal Range and Yungay of 0.2, 0.1, and 0.4% respectively. These values also matched those reported by Robinson et al. (2015) on halites samples colonized by cyanobacteria and heterotrophic species collected at different sites of the hyperarid core of the Atacama, including Yungay, all with less than 0.7%.

## How is Life Able to Survive in the Hyperarid Core with Such Meager Carbon Sources?

Dissolved and particulate organic carbon are critical components in the global carbon cycle and serve as a primary food sources for all Earth’s ecosystems. When considering the characteristics of the hyperarid core, a number of questions arise that may be brought to the attention of the researchers of the field of carbon science (the objective of this hypothesis paper), leading to a number of testable hypothesis and research directions:

(A) Sources of organic carbon:

- What fraction of the organic carbon detected in soils of the hyperarid core is native and how much comes from external sources? There are no reports that have directly tackled this question. However, Ewing et al. (2007) showed that nitrogen and organic carbon concentrations measured in airborne samples at the hyperarid core are consistent with marine aerosols, suggesting long-term non-anthropogenic depositions. In addition, these authors proposed that atmospheric nitrogen deposition dominates nitrogen inventories, which would also probably be the case for organic carbon.

- If a fraction of the organic carbon comes from external sources, where it comes from and how is transported to the hyperarid core? Wang et al. (2014) did not measure the transport of organic carbon as did Ewing et al. (2007), nevertheless these authors showed that different types of salts (Na^+^, Cl^-^ SO4^2-^, Mg^2+^, Ca^+^, etc.) reach the hyperarid core as seawater droplets and anthropogenic emissions transported by the wind in a west to east direction from the Pacific Ocean. This suggestion coincides with the general wind patterns observed in this area provided by the US National Weather Service’s National Centers for Environmental Prediction’s Environmental Modeling Center.

If so, a carbon-focused set of tramps along a west to east transect across the hyperarid core of the Atacama would confirm the sources and rates of atmospheric depositions of organic carbon coming from the Pacific Ocean.

*Hypothesis N1: The main fraction of organic carbon in the soils of the hyperarid core come transported by the winds coming from the Pacific Ocean.*

- What are the native sources of organic carbon? As the Atacama has been an extremely arid place for a long time, most of the native organic carbon in the hyperarid core probably comes from the few microbial species that were able to adapt to its soils million of years ago. This possibility agrees with recent findings of very ancient microorganisms in the Coastal Range of the Atacama (Azua-Bustos et al., 2009), closely related to aquatic species (Azua-Bustos et al., 2012b) thus endorsing a west to east colonization of the hyperarid core by species that migrated with the prevailing winds.

*Hypothesis N2: The native organic fraction in soils of the hyperarid core of the Atacama Desert come from ancient native microorganisms.*

- What fraction of the native organic carbon detected in soils of the hyperarid core represents living soil biota and dead biotic material? Fletcher et al. (2011) directly approached this question using several molecular methods, and concluded that it was difficult to determine whether organic carbon in soils of the hyperarid core comes from dead or living cells, as different methods underestimate or overestimate cell concentrations. Nevertheless, Ewing et al. (2007), suggested that phospholipid fatty acid analysis (PLFA) may be a proper method to solve this question, as PLFA likely represent only viable biomass even at the driest sites.

*Hypothesis N3: The native organic fraction in soils of the hyperarid core of the Atacama Desert can be determined by phospholipid fatty acid analysis.*

(B) Persistence of the organic carbon:

- For how long is organic carbon able to persist in surface soils of the hyperarid core given the presence of oxidizing agents like perchlorates and the continuous exposition to high levels of UV radiation? The Hyperarid core of the Atacama is well known for the widespread presence of a variety of salts, including highly oxidizing species like iodates, chromates and the largest natural deposit of perchlorates (ClO_-4_) known on Earth (Ericksen, 1981). In the hyperarid core of the Atacama, perchlorate concentrations can reach up to 0.6 wt % (Ericksen, 1981), which origin is probably atmospheric (Bao and Gu, 2004; Catling et al., 2010). As already mentioned, Navarro-González et al. (2003) determined the presence of strong oxidants in soils of the Yungay region, but without identifying them. Later, Valdivia-Silva et al. (2009) showed that organics mixed with hyperarid soils are oxidized to CO_2_, suggesting the presence of at least two unidentified types of oxidants; a thermolabile highly oxidative oxidant and a thermostable oxidant with minor oxidative activity. Quinn et al. (2005) also approached this question by characterizing the oxidizing agents present in soil samples of the region of Yungay. These authors found that NO_x_, SO_2_ and O_3_ are oxidized through gas-phase reactions into sulfuric acid and nitric acid, which then adsorb onto aerosols and deposit as dust particles transported by the wind. The levels of these acids in the dust are not high, but their dissolution on the soil surface affect the soil pH which can get highly acidic during periods of higher relative humidity, which in the case of Yungay, are frequent at night time.

Quinn et al. (2007) then directly measured the decomposition of organic compounds by adding ^13^C-labeled alanine, formate and glucose solutions to Yungay soils. These authors reported that during the first days of incubation alanine and glucose decomposed at rates of 0.1–0.2 μmol/d, with formate decomposing at rates of 0.4 μmol/d. These authors also observed equal ^13^CO2 production rates by soils treated with D-glucose and L-alanine (compared to soils treated with L-glucose and D-alanine) suggesting a non-biological chemical decomposition. Interestingly, an increase in the decomposition rates of D-glucose and L-alanine (compared to L-glucose and D-alanine) was later observed, suggesting a second phase of biological decomposition of the added organics.

Pertaining the effects of the UV radiation, there are several reports on the level of tolerance by different species of microorganisms to UV radiation, both native of the Atacama and also introduced as models (Cockell et al., 2008; Paulino-Lima et al., 2013, 2016). However, there are no reports on how specific organic molecules degrade in time when exposed to the daily fluxes of UV radiation typical the hyperarid core of the Atacama. Thus, any organic compound that arrives to the soil surface will most likely be quickly degraded by oxidizing salts and UV radiation, while subsurface organic compounds will be mainly degraded by oxidizing salts. An experiment using different types of organic molecules to measure how long this degradation process takes and what are the products of such degradation will be informative on this point.

*Hypothesis N4: Organic compounds that arrive to the soil surface of the hyperarid core of the Atacama Desert are quickly degraded by oxidizing salts and UV radiation.*
*Hypothesis N5: Organic compounds present below the surface of the soils of the hyperarid core of the Atacama Desert are mainly degraded by oxidizing salts.*

- Does the origin of the organic carbon (external/native) influence its range of persistence in soils of the hyperarid core? As mentioned before, several species of microorganisms highly tolerant to UV radiation have been found in the hyperarid core of the Atacama (Paulino-Lima et al., 2013, 2016), and may be assumed that most of the organic compounds of such species should persist for a longer time in these soils. If so, the organic compounds found in these hyperarid soils should mainly come from native microorganisms, also supporting hypothesis N2.

- Independent of its source, is organic carbon able to move along the soil column of the hyperarid core, how and at what rates?

Given the previous remarks, little or no exogenous organic compounds should be able to move along the soil column at the hyperarid core of the Atacama, as for this not only these compounds should be able to survive the processes of oxidation and UV at its surface, but also would depend on enough rains to move down column (the interaction of organics, oxidizing salts and water have not yet been approached in these sites). A similar assertion would apply for the native organic compounds already present in the soil profile. Altogether this may explain the extremely low organics typically reported in the hyperarid core of the Atacama. However, this interpretation does not answer on how these organic compounds got into the soil depth in the first place.

(C) Metabolism of the organic carbon:

- How are heterotrophic microorganisms able to mobilize and incorporate soil organic carbon given the extremely low amounts of water availability typical of the hyperarid core of the Atacama? This is an extremely interesting question, and up to date there have been no approaches in this subject. It may be assumed that the microorganisms close enough to the soil surface may use the meager amounts of water that is able to infiltrate in the extremely infrequent rain events in this region, and that under these conditions they may access the organic compounds in the close vicinity. An additional possibility is that some of the salts typically present in the hyperarid core of the Atacama are known to be highly hygroscopic, and that through deliquescence they may absorb water under infrequent conditions of high relative humidity (Davila et al., 2008; Robinson et al., 2015). However, this process would only work in the first centimeters of the soil surface, and would not impact the microorganisms present beyond 15 cm of depth. This again leaves the question open on what is the source of water of the microorganisms deeper in the soil profile.

- Can inorganic carbon be used by soil heterotrophic microbial species of the hyperarid core? It is well known that autotrophic nitrifiers are able to reduce inorganic carbon to form organic carbon (Kindaichi et al., 2004). Nitrifying bacteria have been reported in the hyperarid code of the Atacama (Cameron et al., 1966; Ericksen, 1981), thus providing a mechanism on the use of inorganic carbon by heterotrophic bacteria. If so, a bacterial consortia may be expected to be found in the hyperarid core of the Atacama containing the aforementioned metabolic routes.

*Hypothesis S6: Heterotrophic microbial communities present in the soils of the hyperarid core of the Atacama Desert are able to use inorganic carbon with the aid of native nitrifying bacteria.*

Overall, a similar set of core questions are found in field of carbon research, as carbon science explores how much carbon is in Earth, how it moves, what form it takes, where and how it originated, and how it has changed over billions of years. Similarly to the case of carbon research, in order to answer these proposed questions an interdisciplinary approach will be required, integrating the knowledge of chemists, physicists, geologists, and biologists in the case of the hyperarid core of the Atacama Desert.

## Author Contributions

AA-B, CG-S, and GC wrote and reviewed the submitted manuscript.

## Conflict of Interest Statement

The authors declare that the research was conducted in the absence of any commercial or financial relationships that could be construed as a potential conflict of interest.
